# Perioperative anaesthetic management in cytoreductive surgery (CRS) with hyperthermic intraperitoneal chemotherapy (HIPEC): a retrospective analysis in a single tertiary care cancer centre

**DOI:** 10.1515/pp-2022-0001

**Published:** 2022-05-30

**Authors:** Raghav Gupta, Nishkarsh Gupta, Prashant Sirohiya, Anuja Pandit, Brajesh Kumar Ratre, Saurabh Vig, Swati Bhan, Ram Singh, Balbir Kumar, Shweta Bhopale, Seema Mishra, Rakesh Garg, Sachidanand Jee Bharati, Vinod Kumar, Suryanarayana Deo, Sushma Bhatnagar

**Affiliations:** Department of Onco-anesthesia and Palliative Medicine, AIIMS, New Delhi, India; Department of Surgical Oncology, All India Institute of Medical Sciences, New Delhi, India

**Keywords:** anaesthesia, cancer, cytoreductive surgery, fluid therapy, hyperthermic intraperitoneal chemotherapy, perioperative care

## Abstract

**Objectives:**

Cytoreductive surgery (CRS) with hyperthermic intraperitoneal chemotherapy (HIPEC) is associated with increased morbidity and mortality. We retrospectively analysed the perioperative anesthetic management in patients undergoing HIPEC surgery.

**Methods:**

After ethics approval, we reviewed the records of patients who underwent CRS/HIPEC from 2015 until 2020. We noted the peritoneal carcinomatosis index (PCI), blood loss, anastomoses done, total amount of fluid given, delta temperature and duration of surgery. These were correlated with the need for postoperative ventilation, length of ICU stay, Clavien–Dindo score and 30 day mortality.

**Results:**

Of the 180 patients reviewed, the majority were women (85%) with a mean age of 48 years who had ovarian tumors (n=114). The total amount of fluid given was associated with an increased length of ICU stay (p=0.008). Prolonged surgery resulted in increased length of ICU stay (p<0.001), need for postoperative ventilation (p=0.006) and a poor Clavien–Dindo score (p=0.039). A high PCI score correlated with increased ICU stay, 30 day mortality (p<0.001), and the need for postoperative ventilation (0.005).

**Conclusions:**

PCI, duration of surgery and blood loss were major predictors of postoperative morbidity. Additionally, the amount of fluid given and delta temperature affected patient outcome and should be individualized to the patient’s needs.

## Introduction

Peritoneal carcinomatosis (PC) represents a widespread metastatic dissemination throughout the abdomen and pelvis of many organ-based malignancies, particularly carcinomas of the gastrointestinal tract and ovaries. Since the 1980s, cytoreductive surgery (CRS) with hyperthermic intraperitoneal chemotherapy (HIPEC) has been indicated as a treatment for these neoplasms. It consists of almost complete removal of the peritoneal surface, multiple visceral resections, and a variable number of intestinal anastomosis, followed by perfusion of chemotherapy inside the abdominal cavity for 90 min at 42 °C [1, 2]. The wide extent of resection and physicochemical trauma of HIPEC alters capillary permeability and leads to tissue damage. Additionally, CRS is associated with a significant fluid shift attributable to suboptimal nutritional status, prolonged preoperative starvation, intraoperative blood and fluid loss, pharmacological vasodilation by neuraxial and systemic anaesthetic drugs, and vasodilation due to a systemic inflammatory response to surgery (SIRS). This results in hypotension and altered haemodynamics in peri-operative settings. Therefore, rigorous hemodynamic monitoring and appropriate fluid therapy are needed. A permissive infusion regimen has been proposed in the past [3, 4] to counteract fluid, blood, and protein losses; however, it exposes the patient to the risk of fluid overload and tissue oedema. A restrictive infusion regimen may expose the patient to hemodynamic instability, affect tissue perfusion and potentiate the nephrotoxic effect of the chemotherapy drugs. There is strong evidence supporting a goal-directed approach (using the Flotrac/Vigileo system) to perioperative fluid therapy in cases of major abdominal surgeries, leading to a significant reduction in systemic complications [5], [6], [7]. It has also been noted that procedure duration and peritoneal carcinomatosis index (PCI) play a significant role in overall outcome. These surgeries are often associated with complications, increased ICU stay, prolonged hospitalisation time and increased postoperative morbidity (22–41%) and mortality (2–5%) [8, 9]. Additionally, higher PCI, longer duration of surgery, higher delta temperatures, increased blood loss, high intraoperative fluid requirement, lower mean arterial pressure and higher blood product requirement were associated with >24 h postoperative ventilation as well as ICU stay >5 days. There is limited literature on anaesthetic techniques and outcomes following CRS/HIPEC from the developing world. In this study, we retrospectively analysed the effect of perioperative anesthetic variables during HIPEC surgery on overall outcomes.

## Materials and methods

### Study design

The study was performed as per the World Medical Association Declaration of Helsinki. After Ethics Committee approval (IECPG-536/23.09.2020), the records of patients > 18 years who underwent CRS and HIPEC surgery at a tertiary care center for peritoneal surface malignancies and pseudomyxoma peritonei from January 2015 until July 2020 were analysed retrospectively. Patients undergoing staged procedures, those requiring emergency re-exploration and those with incomplete data were excluded.

### Data collection and statistical analysis

These patients were reviewed for demographic parameters (sex, age, diagnosis, body mass index, comorbidities, preoperative treatment history and ASA-PS grading). The details of the intraoperative anaesthesia technique used, vital parameters, total fluid and blood given in three different phases (CRS, HIPEC and post-HIPEC phase), urine output, blood gas analysis, inotropic and chemotherapeutic agent used, number of anastomoses performed and PCI score were reviewed. Postoperative urine output in 24 h, timing of extubation, length of ICU stay, kidney function tests, acute kidney injury (AKIN) classification, modified Clavein–Dindo classification and 30 day mortality were also noted. The perioperative fluid therapy, duration of surgery and PCI were correlated with peri-operative lactate levels, length of ICU stay, AKIN classification, modified Clavein–Dindo classification and 30 day mortality. The effect of delta temperature (maximum temperature during the HIPEC phase – minimum temperature during CRS) on the amount of blood loss intraoperatively, the use of inotropes intraoperatively and coagulation parameters on postoperative days 0, 1 and 2 was also analysed. Pearson’s and Spearman’s correlation coefficients were used to determine the strength of association.

## Results

**Figure 1:**
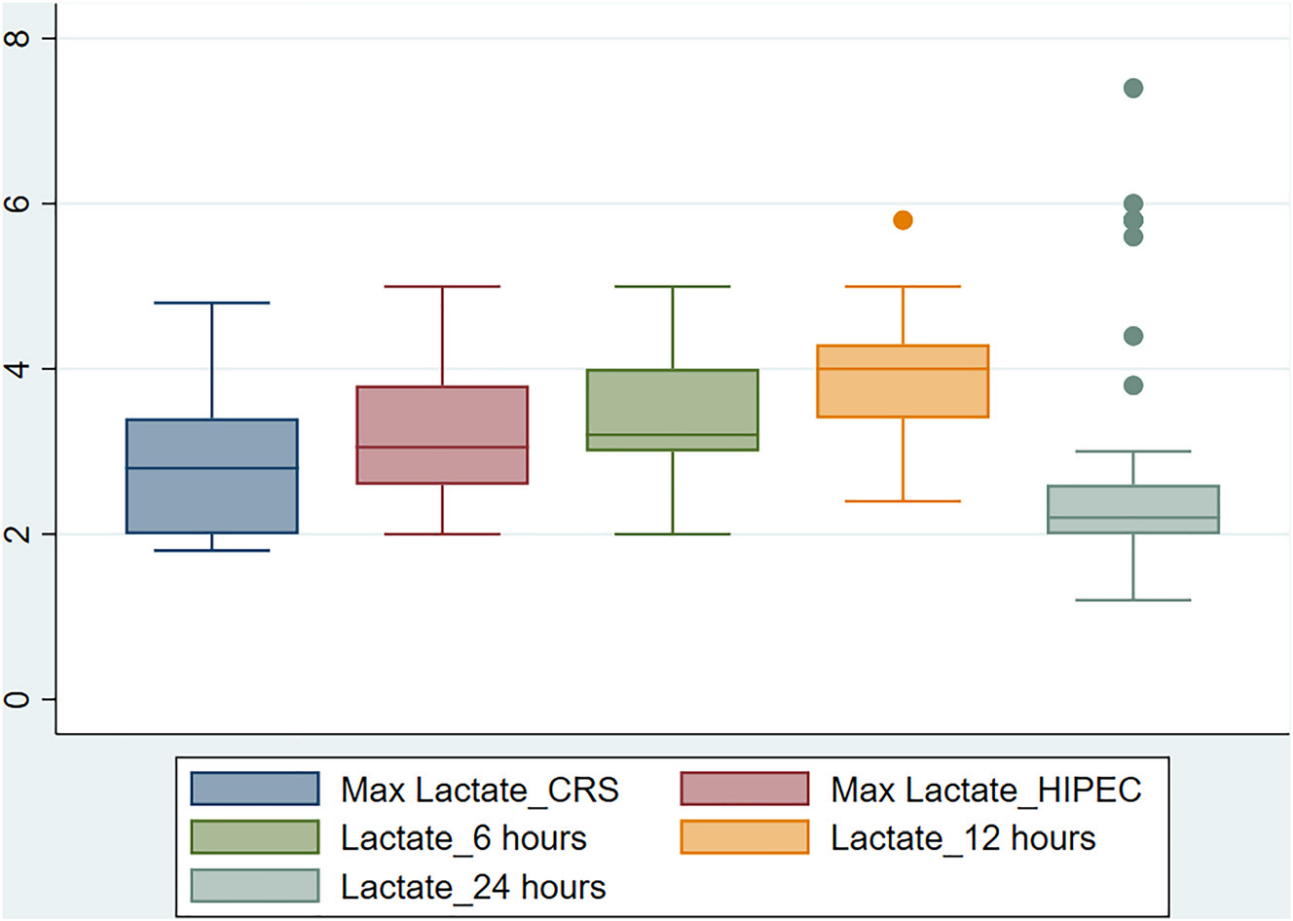
Intra-operative and post-operative course of lactate (Box Plot).

We reviewed the records of 205 patients and excluded 25 patients because of incomplete data. Data were analysed for 180 patients, of whom the majority were women (85%) with a mean age of 48 years who had ovarian tumors (n=114). A total of 74 (41.11%) patients belonged to ASA I, and 101 (56.11%) patients belonged to ASA II. Overall, 48 (26.66%) patients suffered from hypertension, and 30 (16.66%) had diabetes mellitus. Ninety-six (53.33%) out of 180 patients received both neoadjuvant chemotherapy and adjuvant chemotherapy. Preoperative albumin was 3.83±0.81 g/dL, and PCI was 15.76±5.15. Table 1 highlights the perioperative variables of patients undergoing CRS/HIPEC (n=180). In 176 (97.77%) patients, a combined general and regional anaesthesia technique was used. Cisplatin (86%) and mitomycin (9%) were primarily used as chemotherapeutic agents. Figure 1 highlights the maximum lactate level during the CRS phase and HIPEC phase and postoperatively at 6 h, 12 h and 24 h.

**Table 1:** Peri-operative variables of patients undergoing CRS/HIPEC (n=180).

Diagnosis	Day of extubation, days, mean (SD)	Length of ICU stay, days, mean (SD)	Blood loss, mL, mean (SD)	Total IV fluid, mL, mean (SD)	Duration of surgery, hours, mean (SD)	Preoperative albumin, g/dL, mean (SD)	PCI, mean (SD)
Carcinoma (Ca) Ovary (n=114)	1.42 (0.50)	3 (1.26)	502.38 (260.99)	4,057.14 (1,076.37)	6.61 (1.03)	3.90 (0.77)	16 (3.89)
Ca colon (n=21)	1.64 (0.74)	3.07 (0.99)	775 (445.06)	4,678.44 (1,340.59)	6.46 (1.33)	3.49 (0.66)	18.28 (4.21)
Pseudomyxoma (n=14)	1.58 (0.79)	3.33 (2.14)	779.16 (885.60)	4,875 (1,553.95)	6.25 (1.01)	3.49 (0.66)	17.33 (7.46)
Peritoneal mesothelioma (n=12)	1.41 (0.66)	3.16 (1.85)	550 (350.32)	4,083.33 (1,144.81)	6.45 (0.89)	4.06 (0.81)	15.75 (8.86)
Ca appendix (n=12)	1.42 (0.53)	3 (1.15)	442.85 (151.18)	4,071.42 (534.52)	6.78 (0.80)	4.04 (0.93)	17.28 (2.62)
Ca rectum (n=7)	1.42 (0.53)	3 (1.15)	442.85 (151.18)	4,071.42 (534.52)	6.78 (0.80)	4.04 (0.93)	17.28 (2.62)
p-Value	0.88	0.91	0.55	0.36	0.41	0.03	0.16

The duration of surgery was 6.35±1 h, with an average blood loss of 610±445.06 mL. A total of 81 patients out of 180 patients underwent bowel anastomoses. Of these 81 patients, 48 and 33 patients had anastomoses before and after HIPEC, respectively. A total of 97 anastomoses were performed with a mean of 0.70 anastomoses per patient (range 0–2). The total IV fluid given was 4,294.44±1,161.66 mL. The mean urine output during CRS was 234.66±111.48 mL, during HIPEC was 167.66±44.59 mL and during the post-HIPEC phase was 93.94±26.56 mL. The postoperative urine output in the first 24 h after surgery was 793.61±278.16 mL. All patients were shifted to the ICU, and the mean length of ICU stay was 1.43±0.58 days.

### Correlation association between total fluid and different perioperative parameters

There was a negative correlation between total fluid given and serum lactate levels during the CRS/HIPEC phase and at 6, 12 and 24 h post-surgery, which was statistically significant during the CRS/HIPEC phase and at 6 h post-surgery. A positive correlation was observed between total fluid given and length of ICU stay, and this was also statistically significant (Table 2). There was no association between total fluid given with AKIN classification and 30 day mortality.

**Table 2:** Correlation between total fluid and different perioperative parameters.

Variable	Mean(SD) {Median}	Range (min–max)	Pwcorr	p-Value
Total fluid, mL	4,294.44(1,161.66)	2,500–9,500		
	{4,200}			
Lactate_CRS, mmol/L	2.82(0.76)	1.8–4.8	−0.44	**<0.001**
	{2.8}			
Lactate_HIPEC mmol/L	3.18(0.72)	2–5	−.45	**<0.001**
	{3}			
Lactate_6 h mmol/L	3.38(0.56)	2–5	−.21	**0.003**
	{3.2}			
Lactate_12 h mmol/L	3.92(0.51)	2.4–5.8	−.011	0.13
	{4}			
Lactate_24 h mmol/L	2.40(0.80)	1.2–7.4	− 0.02	0.71
	{2.2}			
Length of ICU stay, days	2.89(1.30)	1–8	0.19	0.008
	{3}			

The bold values in the Table highlight that the p-values are statistically significant.

### Correlation and association between the duration of surgery and different perioperative parameters

There was a positive correlation observed between the duration of surgery and the length of ICU stay, which was statistically highly significant (Table 3). There was an association observed between the duration of surgery and Clavien–Dindo classification, which was statistically significant (p-value=0.039), with 7 cases having CD grade >IIIa. Additionally, there was an association between the duration of surgery and the day of extubation (p-value=0.006).

**Table 3:** Correlation between duration of surgery and length of ICU stay.

Variable	Mean (SD)	Range (min–max)	pwcorr	p-Value
Duration of surgery, h	6.35 (1.00)	4.5–10		
Length of ICU stay, days	2.89 (1.30)	1–8	0.47	**<0.001**

The bold values in table highlight that the p-values are statistically significant.

### Correlation and association between PCI and different perioperative parameters

There was a positive correlation between PCI and length of ICU stay, which was statistically significant (Table 4). There was an association observed between PCI and the day of extubation (p-value=0.005), CD grading (p-value=0.001) and 3 day mortality (p-value=0.001).

**Table 4:** Correlation between PCI and length of ICU stay.

Variable	Mean (SD)	Range (min–max)	pwcorr	p-Value
PCI	15.76 (5.15)	1–31		
ICU stay	2.89 (1.30)	1–8	0.72	**<0.001**

The bold values in table highlight that the p-values are statistically significant.

### Correlation and association between the number of anastomoses and different perioperative parameters

No association was observed between the number of intestinal anastomoses and length of ICU stay and 30 day mortality.

### Correlation between delta (Δ) temperature and different perioperative parameters

There was a positive correlation between Δ temperature and intraoperative blood loss, inotrope requirement and PT/INR values on postoperative days 0, 1 and 2, which was statistically significant (Table 5).

**Table 5:** Correlation between delta (Δ) temperature and different perioperative parameters.

Variable	Mean (SD)	Range (min–max)	Spearman’s rho	p-Value
Δ Temperature, °C	2.50 (0.73)	1.4–4.2		
Blood loss. mL	610 (445.06)	200–3,500	0.72	**<0.001**
Inotrope_ CRS, mcg/kg/min	0.14 (0.22)	0–1	0.60	<0.001
Inotrope_ HIPEC, mcg/kg/min	0.19 (0.22)	0–1	0.53	**<0.001**

Variable	Mean (SD)	Range (min–max)	pwcorr	p-Value
PT/INR_POD 0	1.11 (0.15)	0.8–1.4	0.48	**<0.001**
PT/INR_P0D 1	1.15 (0.15)	0.8–1.5	0.52	**<0.001**
PT/INR_P0D 2	1.16 (0.14)	0.7–1.5	0.47	**<0.001**

The bold values in table highlight that the p-values are statistically significant.

### Correlation and association between intraoperative blood loss and different perioperative parameters

There was a positive correlation between blood loss and length of ICU stay, which was statistically significant (Table 6). Additionally, there was an association between blood loss and the day of extubation (p-value <0.001); 109 patients were extubated on day 1, but no association was observed between blood loss and the Clavien–Dindo classification (p-value=0.14).

**Table 6:** Correlation between blood loss and length of ICU stay.

Variable	Mean (SD)	Range (min–max)	Spearman’s rho	p-Value
Blood loss, mL	610 (445.06)	200–3,500		
Length of ICU stay, days	2.89 (1.30)	1–8	0.37	**<0.001**

The bold values in table highlight that the p-values are statistically significant.

## Discussion

In this study, we retrospectively analysed the preoperative, intraoperative and postoperative courses of 180 patients who underwent cytoreductive surgery with hyperthermic intraperitoneal chemotherapy (CRS/HIPEC) over a period of five years. Specific perioperative concerns for CRS/HIPEC highlighted in this study were related to fluid management, duration of surgery, peritoneal carcinomatosis index, delta temperature (maximum temperature during HIPEC phase – minimum temperature during CRS) and blood loss. The present study is one of the largest to specifically assess perioperative anaesthesia-related factors in terms of overall management.

The majority of patients in our study had CA ovaries (63.3%), which is in contrast to the study by Kajdi et al. in which CA appendix (57.8%) was most common [10].

The presence of comorbidities is a common occurrence in cancer patients because of immunocompromised status. The most common comorbidity observed in our study was hypertension, followed by diabetes mellitus and hypothyroidism. Previous studies have also reported hypothyroidism, diabetes, hypertension and ischemic heart disease in these patients [11, 12].

Patients undergoing CRS/HIPEC have a large laparotomy incision and should be provided with good perioperative analgesia. Primary opioid-based analgesia should be used with caution, as it may be associated with increased respiratory complications and may increase the need for ventilator support. Epidural provides good analgesia but is associated with an increased risk of hemodynamic disturbance due to extensive surgery, and these patients are prone to develop spinal hematoma due to coagulation abnormalities and thrombocytopenia. Therefore, the coagulation abnormalities should be assessed and documented to be normal before the insertion and removal of the epidural catheter and ensure that the procedure is atraumatic and performed by experienced anaesthesiologists to avoid complications [13]. Multimodal analgesia with a combination of local anesthetics and opioids in thoracic epidural and intravenous NSAIDs and opioids provides excellent analgesia. This plays an important role in early ambulation, early extubation and decreased postoperative ileus. The majority of patients (97.7%) in our study received combined general and regional anesthesia. This is similar to a study in Switzerland in which they used a combination of GA with RA in 79% of cases [10].

Common chemotherapeutic drugs used during CRS/HIPEC are cisplatin, mitomycin C, doxorubicin and oxaliplatin. These are associated with various side effects, such as nephrotoxicity, neurotoxicity, cardiotoxicity and electrolyte disturbances. C isplatin was the most common chemotherapeutic agent used in our study, followed by mitomycin C. This is in contrast to the study by Kajdi et al. in which they used a combination of chemotherapy drugs (doxorubicin and mitomycin) as the most common. The chemotherapy agent used depends upon the tumour origin, and considering ovarian cancer followed by peritoneal malignancy, cisplatin (50 mg/m^2^ in 1 L of normal saline) and mitomycin C (10–12.5 mg/m^2^ in 1 L of normal saline) were commonly used [11, 12].

During cytoreduction of the tumour, the intraoperative fluid losses may be as high as 8–12 mL/kg and may be associated with significant blood loss depending upon the extent of resection. During the HIPEC phase, saline-enriched chemotherapeutic drugs may increase intra-abdominal pressure, decrease venous return and decrease cardiac output. Since this procedure leads to a massive fluid shift, intraoperative crystalloids and colloids are administered to ensure adequate perfusion pressure and urine output without causing fluid overload. Patients with poor cardiac reserve may not tolerate a high volume of intravenous fluid and may require vasopressors and inotropes to be used judiciously. Various strategies for fluid management have been described, such as “liberal”, “restrictive” or “goal directed”. Liberal fluid administration leads to complications such as fluid overload, surgical site edema and organ dysfunction. Restrictive fluid therapy may hamper kidney perfusion, especially in the setting of hemodynamic fluctuation during the CRS and HIPEC phases. Thus, maintaining optovolemia using goal-directed fluid therapy is the standard of care. Lactate is a surrogate marker of tissue perfusion. Increasing levels of lactate denote tissue hypoperfusion, anaerobic metabolism and increased glucose metabolism [10, 14]. There were increasing levels of lactate found during CRS and the HIPEC phase at 6 h and 12 h postoperatively, which slowly plateaued to normal by 24 h postsurgery. This is similar to the intraoperative course of lactate in a study by Kajdi et al. [10].

Hemodynamics, hyperthermia and the use of cytotoxic chemotherapy during HIPEC increase the risk of renal injury. Intraoperative measurement of urine output is a reliable, noninvasive and surrogate marker of renal perfusion [12]. One should aim at a minimum urine output of 0.5 mL/kg/h during cytoreduction, 2–4 ml/kg/h during the HIPEC phase and 1–2 ml/kg post-HIPEC [15]. Urine output in our study during different phases of surgery, CRS, HIPEC and post-HIPEC, was 234. 6+111.4 mL, 167.6+44.6 mL and 93.9+26.5 mL, respectively. Maintaining targeted urine output is crucial for minimising the risk of acute kidney injury, especially during the HIPEC phase. The diuretic should only be given after ensuring euvolemia and optimal renal perfusion [16].

Patients’ preoperative nutritional status and albumin levels strongly predict the overall length of hospital stay and morbidity [17]. The overall mean albumin level in our patients was 3.83±0.81 g/dL. In the pseudomyxoma group and CA colon group, it was 3.49±0.66 g/dL, which was less than that of the other tumours (p=0.03). This is similar to a study conducted by Kalpana et al. in which they observed mean albumin levels to be the lowest for the pseudomyxoma group because of the aggressive nature of the disease and resection of multiple organs and associated with diaphragmatic stripping in a few cases [11].

In our study, we found a negative correlation between the total amount of fluid infused and intraoperative and postoperative lactate levels during the CRS phase, HIPEC phase and at 6 h postsurgery. This could be due to the maintenance of adequate tissue perfusion by a liberal amount of fluid given. In the landmark study conducted by Myles et al. in which they compared restrictive vs. liberal fluid regimes in major abdominal surgery, they could not find a statistical association for peak lactate levels between the two groups, probably because of missing data [18]. We found a positive correlation between the amount of fluid given and length of ICU stay. This is similar to the study by Kaplana et al. in which they observed longer ICU stays for patients given more fluid [19]. ERAS guidelines for elective major abdominal surgery also recommend the use of a restrictive fluid regime to shorten the length of ICU and hospital stay [20]. No association was found between the total fluid given and 30 day mortality and acute kidney injury in our study, probably because of a goal-directed approach used by us that ensured optimal fluid therapy. This is in contrast to the study by Myles et al. in which they observed that the probability of acute kidney injury was higher in the group with restrictive fluid [21]. Colantonio et al. advocated goal-directed fluid therapy, which decreases morbidity and hospital length of stay and mortality [22]. The numbers studied are small to clearly say if liberal fluid regimen should be avoided but goal-directed fluid management intraoperatively can be routinely used in such massive resections.

A long duration of surgery is usually associated with perioperative hypothermia, coagulopathy and more than expected blood loss. We found a positive correlation and association between the duration of surgery and the length of ICU stay, the day of extubation in the postoperative period and the modified Clavien–Dindo classification [23]. Kalpana et al. also found a positive and significant correlation between the duration of surgery and the need for prolonged postoperative ventilation and ICU stay days [24].

Higher PCI values are a major predictor of postoperative morbidity, especially for scores >30 [9]. Patients with higher PCI values also have more fluid requirements and blood loss associated with it [10]. There was a statistically significant and positive correlation between PCI and the day of extubation, Clavien–Dindo classification, length of ICU stay and 30 day mortality. The mean PCI in our study was 15.76±5.15, with the highest for the CA colon. It is likely that patients with a higher PCI need more extensive resection that would have led to increased blood loss and necessitated more fluid.

Anastomosis of the bowel performed after or before HIPEC does not affect bowel complication rates (leak/perforation) or overall outcome [25]. In another study conducted by Eng et al. they did not find any association between the number of intestinal anastomoses and the comprehensive complication index (CCI) [18]. In our study, we also did not observe any association between the number of anastomoses and length of ICU stay and 30 day mortality.

Extreme temperature fluctuation is very common in these patients. Therefore, body temperature was monitored by two probes. One was placed in the oesophagus for core temperature monitoring, and the other was placed by thermistors present in the inlet and outlet drains of the HIPEC machine to measure the temperature of the abdominal cavity. The increase in delta temperature led to increased intraoperative blood loss, inotropic requirement during CRS and HIPEC phase and postoperative coagulation parameters. The maximum temperature difference occurs in cases with a higher PCI, which leads to a significant decrease in temperature between the baseline and CRS phases as the amount of resection involved increases [11]. Hypothermia is also associated with cardiac morbidity and decreased humoral and cell-mediated immunity [26].

Patients undergoing CRS/HIPEC may have blood loss from a few hundred ml to up to 9,000 mL due to surgical reasons (high disease load) and coagulation abnormalities. Patients who are managed by transfusion of PRBC and crystalloid often develop significant blood loss during the latter part of surgery. Therefore, procoagulant factors (particularly FFP) should be given early to prevent rather than treat coagulopathy. They should be available at the start of surgery itself and administered based on clinical judgement or point of care testing in the form of thromboelastography (TEG) if available. In our study, a significant correlation was observed between the amount of blood loss and the postoperative requirement of ventilation and duration of stay in the ICU. This is similar to a recent review on intensive care outcomes after CRS-HIPEC in which they concluded that the amount of blood loss and blood product requirements affected the ICU length of stay [27]. The strength of the study is that to date, it is one of the largest retrospective analyses. We elucidated multiple perioperative factors and assessed their correlations and associations with the overall outcome of the patients. Additionally, the study was conducted at a dedicated tertiary care centre where CRS/HIPEC surgeries are being conducted regularly. Limitations are that it is a retrospective study; thus, it is difficult to draw a cause-effect relationship between the amount of intraoperative fluid given and postoperative outcomes in terms of length of ICU stay, 30 day mortality and chances of acute kidney injury. It was a study conducted at a single tertiary care cancer centre, and the results may vary in different institutions depending upon the expertise and facilities available. These data spanned a period of five years, during which there have been changes in anaesthetic as well as surgical techniques and the anaesthesia management of patients varied and did not follow a strict protocol.

## Conclusions

In conclusion, this study shows that the peritoneal carcinomatosis index and length of surgery were major predictors of postoperative morbidity in terms of duration of ICU stay, need for postoperative ventilation, poor modified Clavien–Dindo score and 30 day mortality. The total amount of fluid given correlated well with the length of ICU stay. Increased delta temperature was associated with more blood loss, increased inotropic requirement intraoperatively and deranged coagulation profile in the postoperative period. Excess blood loss was positively correlated with the need for postoperative ventilation and length of ICU stay. Thus, CRS/HIPEC is a major surgery with significant morbidity and requires coordinated and concentrated multidisciplinary team involvement, including intensive monitoring of different physiological parameters of the body. Thus, the need of the hour is to develop uniform anaesthetic management plans for patients based on evidence-based medicine and multicentre prospective randomised controlled trials.
